# Mechanically Tunable Composite Hydrogel for Multi-Gesture Motion Monitoring

**DOI:** 10.3390/bios15070412

**Published:** 2025-06-27

**Authors:** Jiabing Zhang, Zilong He, Bin Shen, Jiang Li, Yongtao Tang, Shuhuai Pang, Xiaolin Tian, Shuang Wang, Fengyu Li

**Affiliations:** 1 The Key Laboratory of Intelligent Perception and Image Understanding of Ministry of Education, Xidian University, Xi’an 710071, China; zhangjiabing2013ph@163.com (J.Z.); xltian@mail.xidian.edu.cn (X.T.); 2 The Fourth Medical Center, Department of Orthopedics, Graduate School of Medical School, Chinese PLA General Hospital, Beijing 100853, China; 3 Guangdong Provincial Key Laboratory of Speed Capability Research, College of Chemistry and Materials Science, Jinan University, Guangzhou 510632, China; hezilong0901@stu2022.jnu.edu.cn (Z.H.); binshen@stu2018.jnu.edu.cn (B.S.); lijiang33@stu2020.jnu.edu.cn (J.L.); water@stu2019.jnu.edu.cn (Y.T.); pangshuhuai@stu.jnu.edu.cn (S.P.)

**Keywords:** flexible sensor, PVA hydrogel, mechanically tunable, multi-gesture detection, motion monitoring

## Abstract

Intrinsic conductive ionic hydrogels, endowed with excellent mechanical properties, hold significant promise for applications in wearable and implantable electronics. However, the complexity of exercise and athletics calls for mechanical tunability, facile processability and high conductivity of wearable sensors, which remains a persistent challenge. In this study, we developed a mechanically tunable and high ionic conductive hydrogel patch to approach multi-gesture or motion monitoring. Through adjustment of the ratio of amino trimethylene phosphonic acid (ATMP) and poly(vinyl alcohol) (PVA), the composite hydrogel attains tunable mechanical strength (varying from 50 kPa to 730 kPa), remarkable stretchability (reaching up to 1900% strain), high conductivity (measuring 15.43 S/m), and strong linear sensitivity (with a gauge factor of 2.34 within 100% strain). Benefitting with the tunable mechanical sensitivity, the composite hydrogel patch can perform subtle movement monitoring, such as epidermal pulses or pronounced muscle vibrations; meanwhile, it can also recognize and detect major motions, such as hand gestures. The mechanically tunable composite hydrogel contributes a versatile sensing platform for health or athletic monitoring, with wide and sensitive adoptability.

## 1. Introduction

The rapid development of bioelectronics has spurred significant interest in flexible strain sensors capable of conforming to human skin and enduring mechanical deformation. Diverse applications have been identified for these sensors, encompassing monitoring of human activity and physiology, electronic skin (e-skin), and interactions between humans and machines [1,2,3,4,5]. For such applications, the active materials within strain sensors must possess both electrical conductivity and mechanical flexibility/stretchability. To fulfill these particular needs, a range of designs utilizing active materials have been put forward. Among these, composite materials combining conductivity with stretchability through the percolation of conductive fillers within elastomeric matrices have gained traction for strain sensor applications. As an example, stretchable strain sensors have been created by integrating traditional conductive substances such as metal nanowires and low-dimensional carbon materials into flexible insulating matrices like rubber [6,7,8,9,10,11]. However, achieving composite materials that simultaneously satisfy the demands of electrical and mechanical properties remains challenging due to the inherent trade-off between conductivity and stretchability. Attempts to enhance the conductivity of composite materials by increasing the ratio of conductive fillers inevitably lead to heightened mechanical stiffness or brittleness [12]. To further improve the comfort of flexible strain sensors, the active sensing materials should also possess comparable elastic modulus and mechanical strength. Novel material strategies are necessary to tackle this challenge.

Offering a blend of biocompatibility, high conductivity, and mechanical durability, intrinsic stretchable and conductive materials like ion-conductive hydrogels show great potential as a solution to this problem. These attributes make them highly suitable for diverse bio-compatible applications, including implantable/wearable electronic devices and bioelectronics [13,14,15,16,17,18]. To enhance the stretchability and toughness of ion-conductive hydrogels, various strategies have been explored. Nanocomposite hydrogels and double network hydrogels have emerged as viable approaches for fabricating conductive hydrogels, exemplified by PVA/CNFs hydrogel, reduced graphene oxide/polymer hydrogel, PSS-MUI/gelatin network hydrogel, PEDOT: PSS/DN hydrogel, and PPy-grafted chitosan/poly (acrylic acid) hydrogel [19,20,21,22,23]. Despite these advancements, many hydrogels still exhibit limitations in breaking strength and stretchability post-treatment, while the mismatch in modulus between hydrogels and human skin remains unresolved. There is a pressing need to further enhance the mechanical properties to better match the elastic moduli of soft biological tissue [24]. However, achieving high mechanical strength and ionic conductivity concurrently poses a challenge. Increasing the cross-link density to enhance the mechanical toughness of hydrogels inevitably leads to increased resistance in ion migration [25,26,27]. Additionally, most hydrogel-based sensors are designed for single-channel operation, limiting their ability to detect various human motions simultaneously. Achieving hydrogels with outstanding mechanical characteristics, elevated conductivity, and ease of processing hinges on the strategic formation of a conductive network and the establishment of a cooperative response mechanism tailored to enhance properties based on particular needs. To address these challenges, we introduce a structurally integrated strategy by incorporating aminotris (methylenephosphonic acid) (ATMP) into PVA hydrogels for the first time. Unlike conventional PVA-based hydrogels that rely on NaCl, glycerol, or conductive fillers like MXene or PEDOT:PSS, our design utilizes a single-molecule system where ATMP simultaneously forms hydrogen bonds with PVA and contributes free phosphonic protons for conductivity. This approach achieves tunable mechanical modulus, high ionic conductivity, and wide-range stretchability without the need for additional fillers or complex fabrication processes. A comparative analysis of representative hydrogel systems (Supplementary Table S1) further highlights the performance and simplicity advantages of our method.

**Figure 1:**
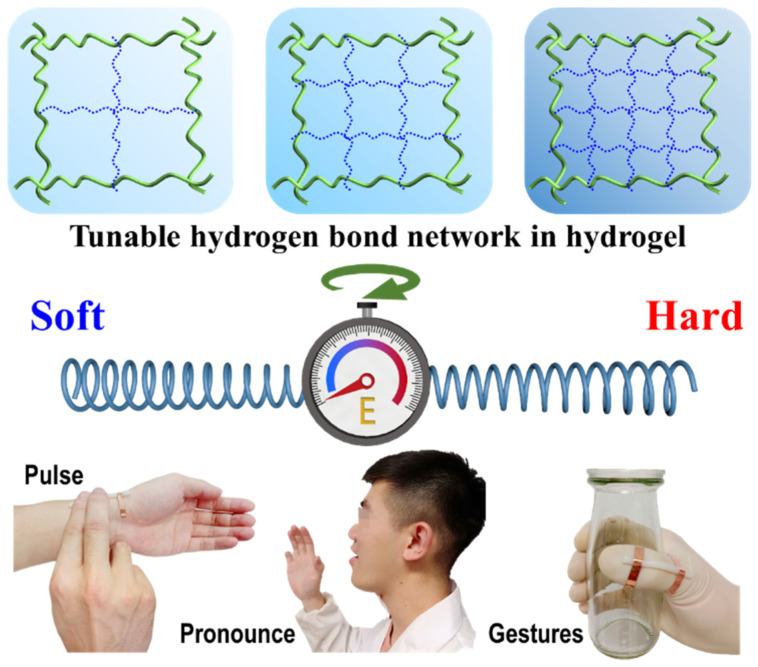
Mechanically tunable hydrogel patch strain sensors for human motion monitoring.

Herein, an ionic conductive hydrogel with tunable mechanical properties was designed and fabricated as a wearable strain sensor (Figure 1). Poly(vinyl alcohol) (PVA) hydrogel was created by blending Amino trimethylene phosphonic acid (ATMP) using a simple freeze–thaw technique. ATMP, abundant in hydroxyl groups, readily forms hydrogen bonds with PVA. Meanwhile, its phosphonic acid groups confer proton conductivity to the resulting PVA-ATMP hydrogel, imbuing it with intrinsic conductivity and mechanical stretchability. Additionally, by controlling the mass ratio of PVA and ATMP, the cross-linking hydrogen bond network within the PVA-ATMP hydrogel can be precisely adjusted, thus allowing for fine-tuning of its mechanical strength and conductivity. This led to the production of hydrogels featuring adjustable mechanical strength (ranging between 50 kPa and 730 kPa), remarkable stretchability (extending to 1900% strain), elevated conductivity (measuring 15.43 S/m), and superb linear sensitivity (with a gauge factor of 2.34 within 100% strain). The remarkable capabilities of the hydrogel patch sensors allow them to demonstrate outstanding effectiveness in detecting both subtle and significant strains related to human movements. This includes the detection of epidermal pulses, which demand high sensitivity, and the recognition of diverse hand gestures, which necessitate wide-ranging stretchability.

## 2. Materials and Methods

### 2.1. Components

A 98% degree of hydrolysis Poly(vinyl alcohol) (PVA) and a 50 wt% solution of Amino tris(methylene phosphonic acid) (ATMP) were procured from Aladdin Chemical Reagent Co., Ltd. (Shanghai, China). Maclin Chemical Reagent Co., Ltd. (Guangzhou, China) supplied Direct blue 86, sodium fluoresces, and rhodamine B. Deionized water underwent purification via a Milli-Q purifier. All remaining chemicals were obtained from Sigma-Aldrich, Ltd. and utilized in their original form.

### 2.2. Fabrication of Devices Utilizing PVA-ATMP Hydrogel

Initially, PVA and ATMP were dissolved in deionized water (the reagent proportions are detailed in Table 1). This mixture was stirred at 105 °C for 2 h to achieve a uniform solution. Subsequently, the solution was cooled to room temperature and poured into molds. After being maintained at −18 °C for 24 h, variously shaped PVA-ATMP hydrogels were obtained. Direct blue 86 was introduced into the PVA-ATMP solution to produce a light blue hydrogel.

### 2.3. Analysis of PVA-ATMP Hydrogel

Using the Frontier instrument (PerkinElmer FTIR spectrometer, manufactured by PerkinElmer Inc., Waltham, MA, USA), the FTIR spectrum of 2 mm film samples was recorded at 20 °C. XRD analysis was performed with a Miniflex 600 instrument (manufactured by Rigaku Corporation, Tokyo, Japan) (Cu Kα radiation, scanning from 2θ = 5 to 60° at a rate of 5° per minute). UV-vis spectra were acquired using a UV759CRT spectrophotometer (manufactured by Shanghai Youke Instrument, Shanghai, China) over the range of 400 to 700 nm, with a resolution of 1 nm and a quartz cuvette substrate.

Hydrogels measuring 60 mm in length, 20 mm in width, and 3 mm in thickness underwent tensile tests at room temperature using an AG-1 mechanical instrument (manufactured by ZEISS, Oberkochen, Germany), with a testing speed of 50 mm per minute. Subsequently, compressive tests were carried out on hydrogels with a diameter of 30 mm and a height of 70 mm using the same AG-1 mechanical instrument, operating at a speed of 10 mm per minute. To ensure signal stability and eliminate transient fluctuations caused by equipment inertia or hydrogel relaxation, each strain or pressure level was maintained for at least 5 s during testing. Additionally, all mechanical–electrical tests were conducted under controlled ambient conditions (25 ± 1 °C, relative humidity ~50%) to ensure data consistency. Each test was repeated three times (n = 3) using independently prepared samples, and the variation in ΔR/R_0_ values was within ±5%, confirming high repeatability. The sensor’s responsivity, indicated by the gauge factor, was determined by the ratio of the normalized resistance change, ΔR/R0, where R0 represents the initial resistance and ΔR denotes the resistance alteration during stretching. Conductivity (σ) measurements were conducted using the LCR meter IM3536 (manufactured by HIOKI E.E. Corporation, Ueda, Japan), with specific calculation procedures outlined as follows:

σ = L/(R·A)(1)

The parameters L, R, and A are defined as the length, resistance, and area of the samples, respectively.

## 3. Results

**Figure 2:**
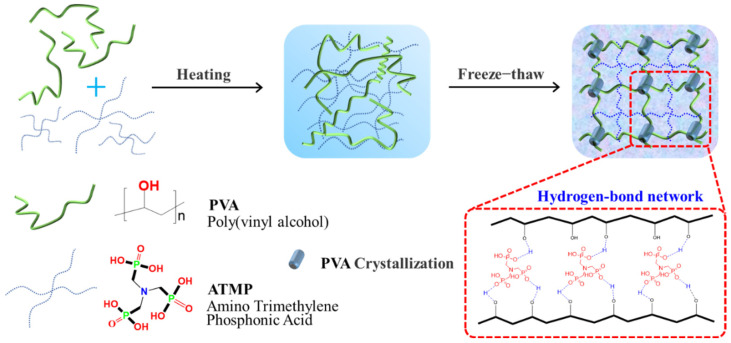
Scheme for the formation of the PVA-ATMP hydrogels after only one freeze–thaw cycle.

We utilized a straightforward one-pot technique to synthesize the water-based PVA-ATMP hydrogel. The hydrogel formation process is illustrated in Figure 2. Initially, homogeneous solutions containing PVA and ATMP at various mass ratios in deionized (DI) water (referred to as PVA-ATMP-15, PVA-ATMP-20, and PVA-ATMP-30) (as specified in Table 1) were left to incubate overnight at −18 °C. Subsequently, the homogeneous sols underwent a rapid freeze–thaw cycle, resulting in the cross-linking of PVA and ATMP via a hydrogen bond network, ultimately producing the PVA-ATMP hydrogel.

**Figure 3:**
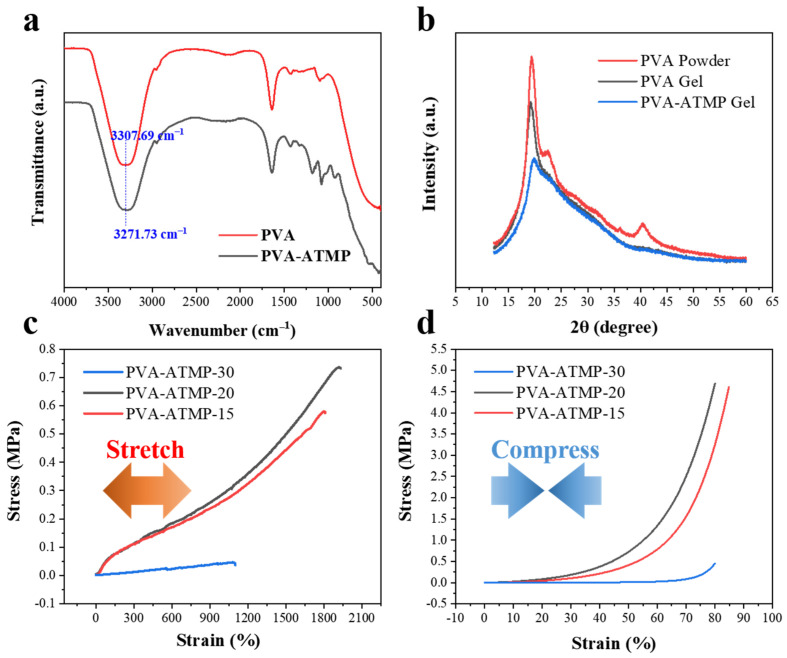
Properties of PVA-ATMP hydrogels. (**a**) Comparative FTIR spectra of pure PVA gel and PVA-ATMP gel. (**b**) XRD patterns of PVA powder, pure PVA gel, and PVA-ATMP gel. (**c**) Stress–strain diagrams illustrating the behavior of hydrogels with different ATMP concentrations. (**d**) Compressive stress–strain profiles of hydrogels with varying ATMP concentrations.

For confirmation of the cross-linking between ATMP and PVA, we conducted a comparison of the FTIR spectra of pure PVA and PVA-ATMP gel. As shown in Figure 3a and Supplementary Figure S2, the characteristic symmetrical stretching vibration of the hydroxyl group in PVA gel appears at 3307 cm^−1^, whereas it shifts to 3271 cm^−1^ in the PVA-ATMP gel. The redshift (~36 cm^−1^) together with the emergence of new absorption bands at ~1076 cm^−1^ (P = O stretching) and subtle shifts in the C-O vibration near 1095 cm^−1^ indicate the formation of hydrogen bonds between PVA and ATMP as well as the chemical incorporation of phosphate groups. These spectral features reflect enhanced hydrogen bonding and modifications to the local electron environment caused by ATMP, confirming the formation of the PVA-ATMP hydrogen. Further confirmation of the formation of the PVA-ATMP hydrogel was obtained through X-ray diffraction (XRD) analysis (Figure 3b). The presence of a lower crystallization peak at approximately 20° in the PVA-ATMP spectrum suggests weakened crystallization of PVA domains due to cross-linking with ATMP via hydrogen bonds. This occurrence boosts the hydrogel’s mechanical robustness. Stretchability and compression tests were conducted to assess the mechanical tunability of the PVA-ATMP hydrogels for practical applications. Representative stress–strain curves of the hydrogels are presented in Figure 3c,d. By altering the mass ratio of PVA to ATMP, we discovered that the mechanical characteristics of the hydrogel could be modified. Results indicated that the tensile modulus, compression modulus, and stretchability of the hydrogels increase with increasing PVA concentration. Compared to PVA-ATMP-15 and PVA-ATMP-30, the tensile modulus and compression modulus of PVA-ATMP-20 increased to 730 kPa and 4700 kPa, respectively. Furthermore, a maximum stretchability of up to 1900% was achieved as the concentration of PVA in PVA-ATMP-20 increased to 11.7 wt%. The adjustable mechanical characteristics observed are ascribed to non-covalent connections, including dynamically disassociated and reassociated hydrogen bonds established between ATMP and PVA molecules. These connections interfere with the interaction among PVA chains, functioning as sacrificial links to efficiently disperse energy prior to the rupture of PVA chains during mechanical distortion.

**Figure 4:**
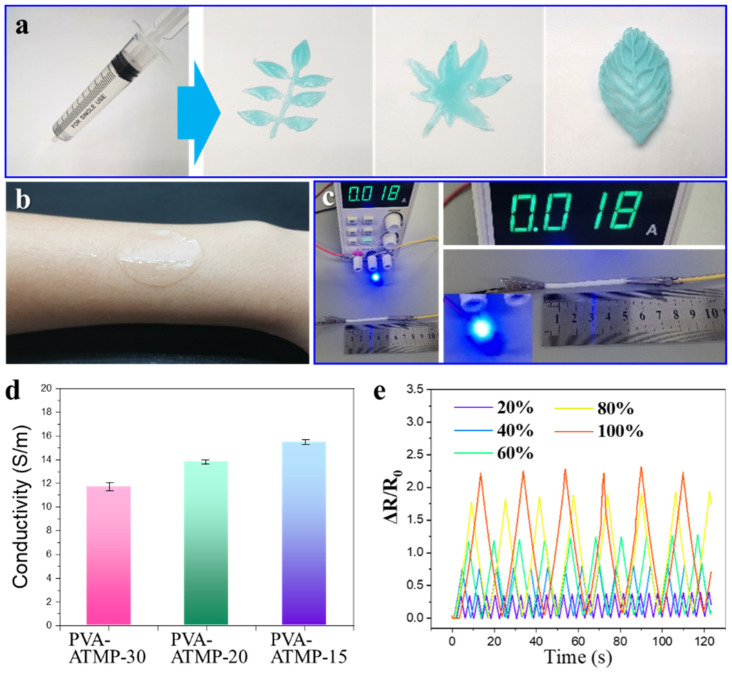
The characteristics of PVA-ATMP hydrogel. (**a**,**b**) Thermoplastic properties of PVA-ATMP hydrogel. (**c**,**d**) Conductivity of PVA-ATMP hydrogel. (**e**) The fatigue resistance performance of PVA-ATMP hydrogel.

Ease of processing is a highly sought-after characteristic for hydrogels intended for use in flexible/wearable sensors. As depicted in Figure 4a, the mixture of PVA and ATMP sol can be readily poured into preferred three-dimensional molds or extruded using a syringe to create diverse patterns. The elastomeric nature of hydrogel enables it to achieve conformal contact with the skin. Following this, the conductive characteristics of the PVA-ATMP hydrogel were examined by incorporating the gel into a circuit connected in series with an LED indicator (as illustrated in Figure 4c). In its original state, the PVA-ATMP hydrogel could function as a conductor, illuminating the LED. Remarkably, impressive conductivity was observed in all PVA-ATMP hydrogels with varied loading ratios of ATMP. It is noteworthy that as the ATMP concentration increased, the conductivity of the hydrogels also increased accordingly. Specifically, the PVA-ATMP-30, PVA-ATMP-20, and PVA-ATMP-15 hydrogels demonstrated conductivities of 11.76 S/m, 13.73 S/m, and 15.43 S/m, respectively (Figure 4d). This phenomenon arises from the fact that the conductivity of the hydrogel is governed by proton transfer within the polymer network. Specifically, ATMP contains abundant phosphonic acid (–PO_3_H_2_) and hydroxyl (–OH) groups, which dissociate under hydration to release free protons and form hydrogen-bonded hydration shells. These features facilitate proton hopping through the Grotthuss mechanism, enabling fast ion transport within the water-rich hydrogel matrix. Additionally, the dynamic hydrogen bonds formed between ATMP and PVA chains generate interconnected conduction pathways across the network, further promoting proton mobility and supporting the observed high ionic conductivity. To further verify this mechanism, electrochemical impedance spectroscopy (EIS) analysis was conducted. As shown in Supplementary Figure S3, the PVA-ATMP hydrogel exhibited a smaller semicircular arc and steeper low-frequency tail than the pure PVA hydrogel, indicating a reduced charge transfer resistance and enhanced ion mobility. These results strongly support the involvement of a quasi-Grotthuss proton hopping mechanism enabled by the synergistic effect of phosphonic acid groups and hydrogen bonding interactions. Consequently, the PVA-ATMP-15 hydrogel with the highest ATMP loading ratio exhibited the highest conductivity due to its ability to provide a greater number of freely moving protons. Subsequently, resistance variations in the PVA-ATMP hydrogels were continuously monitored at different strains. As illustrated in Figure 4e, the hydrogels displayed good repeatability in detecting moderate to high strains ranging from 20% to 100%. To further evaluate the multifunctionality of the PVA-ATMP hydrogels, we assessed their optical transparency. As shown in Supplementary Figure S4, the hydrogel exhibits high light transmittance across the 300–700 nm range, with a peak transmittance of 72.1% at 550 nm, suggesting excellent optical clarity for potential applications in transparent and skin-interfacing wearable electronics. In addition, we assessed the hydrogel’s short-term dehydration resistance by monitoring its conductivity and mass loss after 3 h of storage at 25 °C and 50% relative humidity. As shown in Supplementary Figures S5 and S10, the hydrogel retained over 85% of its original conductivity with only 3.85% water loss, demonstrating good environmental stability in open-air conditions.

**Figure 5:**
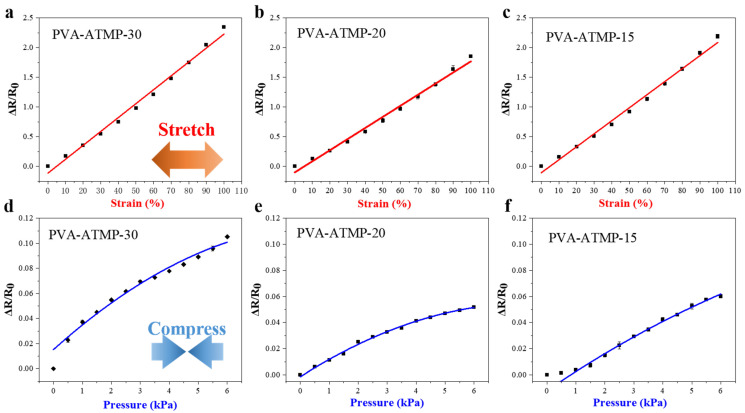
Electrochemical performance of the PVA-ATMP hydrogels with different PVA and ATMP contents. (**a**–**c**) Resistance–strain curves; (**d**–**f**) Resistance–pressure curves.

In addition to their ability to withstand significant deformation, hydrogels intended for use as active materials in strain sensors for E-skin applications should exhibit continuously varying conductivity during stretching/compression, ensuring robust sensitivity across a wide strain range. To assess these properties, the electromechanical features of the PVA-ATMP hydrogels were assessed by monitoring the relative change in resistance, denoted as ∆R/R0, while subjecting them to stretching. Here, ∆R signifies the alteration in resistance in reaction to strain or pressure loading, whereas R0 represents the initial resistance of the sensor in the absence of loading. Figure 5 depicted the strain/pressure curves showing the relative change in resistance, which defines the gauge factor (GF). The inclines of these curves indicate the sensitivity of the sensor. As depicted in Figure 5a–c, all PVA-ATMP hydrogels exhibit a linear region within the stretching range of 0–110%, yielding GF values ranging from 1.87 to 2.34, indicative of high sensitivity. Generally, the deformation of the epidermis during human movement ranges from 0 to 75% [28]. The high GF values observed across the wide detection range of 0–110% suggested that PVA-ATMP hydrogels patched as strain sensors are well-suited for human motion monitoring. Notably, it is worth mentioning that the highest GF value of 2.34 can be achieved in PVA-ATMP-30, as the concentrations of PVA and ATMP decrease. Compared with representative double-network (DN) hydrogels employing covalent sacrificial bonds, our physically crosslinked PVA-ATMP hydrogel achieves superior stretchability (1900%) with excellent self-recovery, as summarized in Supplementary Table S2. This highlights the effectiveness of our dynamic hydrogen bond design in achieving extreme deformation tolerance with structural simplicity and reusability. Furthermore, we investigated the resistance change versus external pressure on the hydrogels (Figure 5d–f), with the characteristic curves indicating that PVA-ATMP-30 exhibits superior sensitivity. This direction-dependent behavior—linear under tension and nonlinear under compression—is attributed to the ordered unfolding of the hydrogen bond network during stretching versus the densification and pathway restriction during compression [29]. To further illustrate the performance enhancement achieved through ATMP regulation, we conducted direct comparisons with unmodified pure PVA hydrogel. As shown in Supplementary Figure S7 and Table S3, the PVA-ATMP hydrogel exhibited significant improvements in modulus (12.25 kPa to 730 kPa), conductivity (0.000934 S/m to 15.43 S/m), and gauge factor (3.78 × 10^−5^ to 2.34), confirming the effectiveness of our hydrogen bond network design strategy. To further contextualize the performance of our hydrogel within the broader landscape of high-performance ionic conductive hydrogels, we conducted a quantitative comparison with representative systems reported in the recent literature. As summarized in Supplementary Table S1, our PVA-ATMP hydrogel outperforms many existing systems in terms of conductivity (15.43 S/m), tunable modulus (up to 730 kPa), and gauge factor (2.34), while maintaining filler-free composition and structural simplicity. This benchmarking underscores the unique integration of mechanical robustness, high sensitivity, and excellent ion conduction in our design.

## 4. Discussion

**Figure 6:**
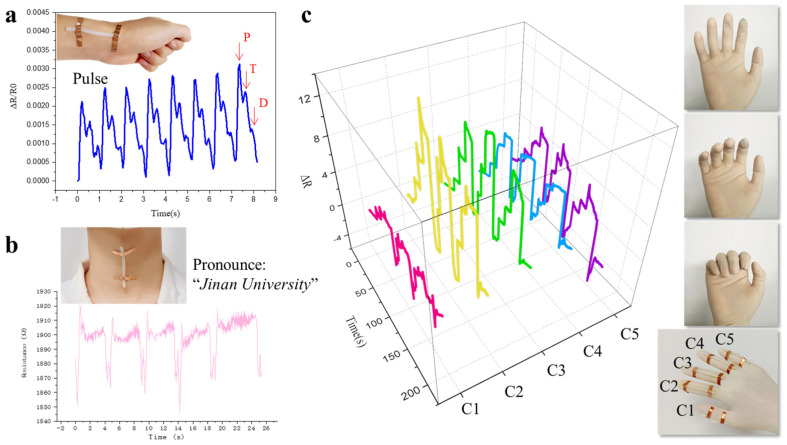
Strain sensing characteristics of the PVA-ATMP hydrogel patch sensors. (**a**) Pulse monitoring. (**b**) Throats pronunciation motion monitoring. (**c**) Five-channel hydrogel patch sensor array for finger bending detection.

Although the gauge factor (GF) of our PVA-ATMP hydrogel is moderate (2.34), compared to some crack-based or filler-enhanced hydrogel systems reporting GF values > 5, it offers substantial practical advantages. As summarized in Supplementary Table S4, many high-GF systems suffer from low reversibility, narrow strain detection ranges, or structural fragility, making them less suited for wearable sensing applications. In contrast, our hydrogel delivers a stable, linear response across a broad strain window (0–110%) with excellent repeatability and mechanical flexibility, which is more compatible with continuous human motion monitoring. With its ease of processing, excellent mechanical tunability, and sensitivity to deformation, the PVA-ATMP hydrogels offer promising avenues for application in detecting subtle and large strains associated with human activities (Figure 6). Initially, we affixed the sensor to the wrist to capture epidermal pulse signals. As depicted in Figure 6a, the pulse waves exhibited three characteristic peaks: percussion wave (P), tidal wave (T), and dicrotic wave (D). Subsequently, we positioned the hydrogel sensor on the throat to investigate its electrical response to minute strains induced by muscle motion during pronunciation, as demonstrated by uttering ‘Jinan University’ (Figure 6b). Analysis of resistance variations facilitated easy detection of pronounce muscle vibration, highlighting the potential for personalized vocal recognition applications. In addition to detecting subtle strains, the hydrogel sensors proved capable of sensing large strains associated with human motion. Following a similar approach used for pulse and voice monitoring, we devised a five-channel hydrogel patched sensor array by attaching five hydrogel sensors to the fingers of the subject’s right hand (Figure 6c). As the fingers bent, the multi-channel sensors responded simultaneously and exhibited reliable monitoring behavior. The resistance of each channel increased proportionally with the degree of finger bending (30°, 60°, and 90°, respectively), underscoring the sensor’s ability to discern different ranges of joint motion. This bending process was repeated three times, with each channel demonstrating consistent responses in the cycling test, affirming the multi-channel sensor platform’s capability to differentiate movement across different fingers. To further substantiate the performance advantages of the PVA-ATMP hydrogel, we conducted control experiments using pure PVA hydrogel under identical testing conditions. As presented in Supplementary Figure S8, the PVA-ATMP hydrogel displayed markedly improved signal clarity and stability during physiological monitoring compared to the pure PVA hydrogel, which exhibited weak and unstable responses. Furthermore, to confirm the real-time repeatability and recognition stability of our sensor, we performed repeated tests using the same PVA-ATMP hydrogel sample. As shown in Supplementary Figure S9, the sensor reliably captured triphasic pulse waves and produced consistent throat across multiple trials, validating its robustness for dynamic physiological sensing applications. These results confirm not only the hydrogel’s electrical and mechanical reliability under dynamic physiological conditions, but also underscore its practical suitability for direct skin contact applications. Importantly, although in vivo biocompatibility tests were not conducted in this study, the intrinsic material design offers strong theoretical support for biological safety. Both PVA and ATMP are widely used biocompatible components; PVA is FDA-approved for medical applications such as artificial skin and wound dressings [30], while ATMP is a hydrophilic, low-toxicity phosphonic acid that forms hydration shells rather than chemically reactive residues. Moreover, the PVA-ATMP hydrogel adopts a purely physical crosslinking mechanism without the use of chemical initiators or toxic additives, which reduces the risk of irritation or immunogenicity [31]. These features suggest that the hydrogel is well-suited for long-term skin-mounted wearable electronics, with minimal risk of adverse biological responses.

In addition, we further evaluated the sensor’s environmental stability, mechanical reversibility, and adaptability to bending deformation. As shown in Supplementary Figures S10–S12, the hydrogel sensor maintained high conductivity and low water loss after air exposure, exhibited stable responses over 200 stretch–release cycles, and responded reliably to bending angles of 30°, 60°, and 90° with fast response times (<240 ms), demonstrating its long-term durability and practical usability for flexible wearable electronics.

**Figure 7:**
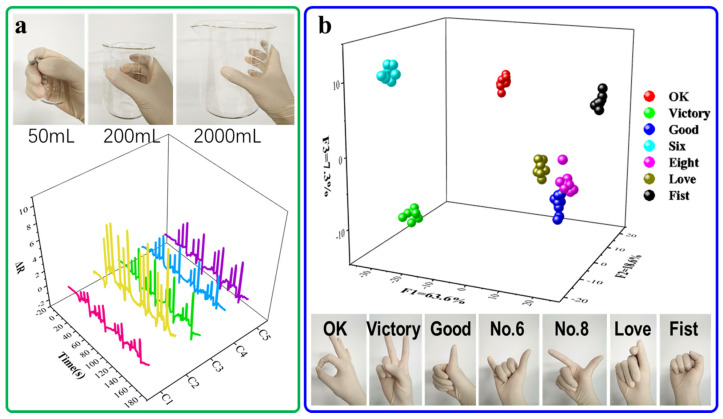
Applications of hydrogel patched as strain sensors. (**a**) Feedback grasping signals for holding different size beakers. (**b**) Hand gesture recognition system for analysis of seven hand gestures.

To clearly demonstrate the practical deployment of the hydrogel sensor for gesture monitoring, we have added Supplementary Figure S13, which illustrates the sensor placement and full system architecture. The PVA-ATMP hydrogel patches were affixed to the second knuckles of five fingers using medical-grade transparent tape, with copper wires connected at both ends for real-time signal acquisition and processing via an Arduino-based system. To further showcase the practical utility of the multi-channel hydrogel patch sensor platform, we initially employed the sensor array as a feedback sensing unit for monitoring finger grasping. Five hydrogel sensors were affixed to the fingers of the subject’s right hand. As illustrated in Figure 7a, during tasks involving grasping beakers of varying sizes, the sensors provided sensitive and reproducible feedback. In the subsequent application, we developed a hand gesture recognition system by integrating the hydrogel patch sensing unit with linear discriminant analysis (LDA). This system was utilized to discern seven hand gestures, including OK, V-sign, thumbs-up, Chinese number hand gestures for six and eight, finger heart, and fist formation (Figure 7b). The LDA facilitated nearly perfect differentiation between features associated with different hand gestures. Beyond conventional gesture recognition, the PVA-ATMP hydrogel sensor also holds significant promise in healthcare-related applications. Potential directions include early detection of neurodegenerative disorders via tremor monitoring [32], real-time rehabilitation tracking [33], non-invasive dysphagia assessment through throat motion analysis, and integration into home-based virtual recovery systems [34]. These prospects highlight the broader clinical and diagnostic relevance of our material platform. Moreover, the sensor exhibited stable performance under wet conditions, including high humidity, short-term immersion, and skin moisture (Supplementary Figures S17–S19), demonstrating good environmental adaptability for potential applications in swimming or humid environments. A PU or PDMS encapsulation is also suggested to further enhance water resistance for long-term underwater applications [35].

## 5. Conclusions

In summary, we developed a mechanically tunable and high ionic conductive hydrogel patch to approach multi-gesture or motion monitoring. A series of ATMP-PVA hydrogels was investigated to attain the combination of mechanical properties and ionic conductivity. Through tunable hydrogen bond networks between ATMP and PVA, the optimized composite hydrogels can approach modifiable mechanical strength (varying between 50 kPa and 730 kPa), outstanding stretchability (extending to 1900% strain), elevated conductivity (measuring 15.43 S/m), and satisfactory linear sensitivity (with a gauge factor of 2.34 within 100% strain). With these attributes, we adopted the hydrogel patches as strain sensors capable of detecting subtle and large strains associated with human activities, such as epidermal pulse, pronunciation muscle vibrations or hand gestures. The mechanically tunable composite hydrogel contributes a versatile sensing platform for health or athletic monitoring with wide and sensitive adoptability.

## Figures and Tables

**Table 1:** The composition of PVA-ATMP hydrogels.

Hydrogel	PVA (g)	ATMP (mL)	DI Water (mL)
PVA-ATMP-15	3	10	15
PVA-ATMP-20	4	10	20
PVA-ATMP-30	4	10	30

## Data Availability

Data will be made available on request.

## Supplementary Materials

The following supporting information can be downloaded at: https://www.mdpi.com/article/10.3390/bios15070412/s1, Figure S1: SEM imageof PVA-ATMP hydrogels. Figure S2: Normalized comparative FTIR spectra of pure PVA gel and PVA-ATMP gel. Figure S3: EIS Spectra of PVA and PVA-ATMP Hydrogels. Figure S4: The UV–Visible Transmittance Spectrum of PVA-ATMP Hydrogel. Figure S5: Short-Term Dehydration Resistance of PVA-ATMP Hydrogel. Figure S6: (a–c) Tensile resistance-strain curves of PVA-ATMP hydrogels with different ratios; (d–f) Corresponding compression resistance—strain curves. Figure S7: Performance Characteristics of Pure PVA Hydrogel. Figure S8: Performance Comparison Between PVA and PVA-ATMP Hydrogels. Figure S9: Real-time repeatability of diverse human physiological signals. Figure S10: Environmental Stability of PVA-ATMP Hydrogel. Figure S11: Cyclic Response of PVA-ATMP Hydrogel under 20% Strain for 200 Stretch–Release Cycles. Figure S12: Dynamic electrical response of PVA-ATMP hydrogel under different bending angles (30°, 60°, 90°). Figure S13: System architecture for sensor-based gesture recognition and analysis. Figure S14: Schematic diagram of circuit design of PVA-ATMP hydrogel sensor for complex action analysis. (a) Series circuit; (b) Parallel circuit. Figure S15: The discriminated analysis of seven hand gestures. Figure S16: HCA score plots for the discrimination of seven hand gestures. Figure S17: Conductivity Variation of PVA-ATMP Hydrogel After 2-Hour Exposure to 90% Relative Humidity. Figure S18: Conductivity Variation of PVA-ATMP Hydrogel After Short-Term Immersion in Water. Figure S19: Strain Response Variation of PVA-ATMP Hydrogel Under Humid Conditions. Table S1: Performance comparison between this work and representative hydrogels. Table S2: Comparison of High-Stretchability DN Hydrogels vs. PVA-ATMP. Table S3: Performance Comparison Between Pure PVA and PVA-ATMP Hydrogels. Table S4: Comparison of High-GF Hydrogel Strain Sensors. Table S5: Results of the LDA of seven hand gestures. Table S6: Comparison of the conductivity and Maximum tensile strength of our Multi-Functional Sensors with Existing ion gel sensors.. References [36,37,38,39,40,41,42,43,44,45,46,47,48,49,50,51,52,53,54,55,56,57,58,59,60,61,62,63,64] are cited in the Supplementary Materials.

## Abbreviations

The following abbreviations are used in this manuscript:

ATMP	Amino trimethylene phosphonic acid
PVA	Poly(vinyl alcohol)
GF	Gauge factor
LDA	Linear discriminant analysis
