# Supplementary material for: Mechanically Tunable Composite Hydrogel for Multi-Gesture Motion Monitoring
Source: Biosensors (Basel). 2025 Jun 27;15(7):412. doi: 10.3390/bios15070412 (PMC12293851; doi:10.3390/bios15070412)
Supplement: Supplementary file 1 [file biosensors-15-00412-s001.zip › biosensors-3659578-supplementary.pdf]

Supplementary Information

Mechanically Tunable Composite Hydrogel for Multi-Gesture Motion Monitoring

Jiabing Zhang[1,2], Zilong He[3], Bin Shen[3], Jiang Li[3], Yongtao Tang[3], Shuhuai Pang[3], Xiaolin Tian[1], Shuang Wang[1,*] and Fengyu Li[3,*]

1 Xidian University, Xi'an, 710071, P. R. China; shwang@ mail.xidian.edu.cn

2 Graduate School of Medical School, Department of Orthopedics, the Fourth medical center, Chinese PLA General Hospital, Beijing,100853, P. R. China;

3 College of Chemistry and Materials Science, Guangdong Provincial Key Laboratory of Speed Capability Research, Jinan University, Guangzhou, 510632, P. R. China; lifengyu@jnu.edu.cn

* Correspondence: shwang@mail.xidian.edu.cn & lifengyu@jnu.edu.cn

Contents

Materials and Methods 2
Data analysis 2
Figures 4
Tables 15
References 18

Materials and Methods

PVA (4 g), ATMP (10 mL) were dissolved in deionized water (30 mL). The mixture was stirred for 2 h at 105 °C to obtain a homogeneous solution. Then, the blended solution was cooled down to room temperature, and formed the sol state of ATMP- PVA. The ATMP- PVA sol was poured into different molds at -18 °C overnight.

The Fourier transform infrared (FTIR) spectrum on 2 mm film samples were recorded on the Frontier instrument at 20 °C. X-ray diffraction (XRD) was carried out with a Miniflex 600 (Cu Kα radiation, 2θ = 5–60 °, scan rate of 5 °·min^{-1}). Ultraviolet-visible (UV-vis) spectra were recorded on an UV759CRT spectrophotometer (Shanghai Youke Instrument) in the wavelength range from 400 to 700 nm, with a resolution of 1 nm and a quartz cuvette as the substrate.The microstructures of the hydrogels were characterized by SEM (ZEISS Supra 40VP). Before character-ization, hydrogel samples were freeze-dried using a freeze-dryer. The freeze-dried hydrogels were exposed to liquid nitrogen to brittlely break the inside and sputtered with gold.

The tensile tests of hydrogels (length of 60 mm, width of 20 mm, and thickness of 3 mm) were carried out on an AG-1 mechanical instrument with a speed of 50 mm·min^{-1} at room temperature. The compressive tests of hydrogels (diameter of 30 mm and height of 70 mm) were implemented on an AG-1 mechanical instrument with a speed of 10 mm·min^{-1}. To ensure signal stability and eliminate transient fluctuations caused by equipment inertia or hydrogel relaxation, each strain or pressure level was maintained for at least 5 seconds during testing. Additionally, all mechanical-electrical tests were conducted under controlled ambient conditions (25 ± 1 °C, relative humidity ~50%) to ensure data consistency. Each test was repeated three times (n = 3) using independently prepared samples, and the variation in $\Delta R/R_0$ values was within ±5%, confirming high repeatability. The responsivity (i.e., gauge factor) of the sensor was tracked by the normalized change of resistance, ΔR/R0, where R0 and ΔR are the original resistance and the resistance change upon stretching, respectively. The conductivity (σ) was recorded with the LCR meter IM3536 and the specific calculation method is as follows:

$$\sigma = L/(R \cdot A)$$

where L, R and A are defined as the length, resistance, and area of samples respectively.

Data analysis

The statistical multivariate methods, linear discriminant analysis (LDA) and principal component analysis (PCA) are routinely used to interpret and evaluate the responses from hydrogel sensor, providing a graphical output to gain an insight of the response data, and calculate classification accuracy. [1-2] The LDA was carried out using SYSTAT® v12.02.00 and the PCA was carried out using Minitab® v16.1.1.0.

Linear discriminant analysis (LDA) of single analyte: A complete way LDA was used including all variables in the analysis. A cross-validation (leave-one-out) routine was used to test the predictability of the sensor by leaving one observation of the set out at the time and uses the rest of the data as a training set to generate the linear

discriminant function (Table 1). Furthermore, LDA generates a new reduced space, from the three-dimensional space generated by the responses of the sensor, defined by the canonical roots (factors or discriminant axes). Each response data group with several repetitions can be reduced to a single score and plotted in the new canonical space (canonical score plot).

Figures

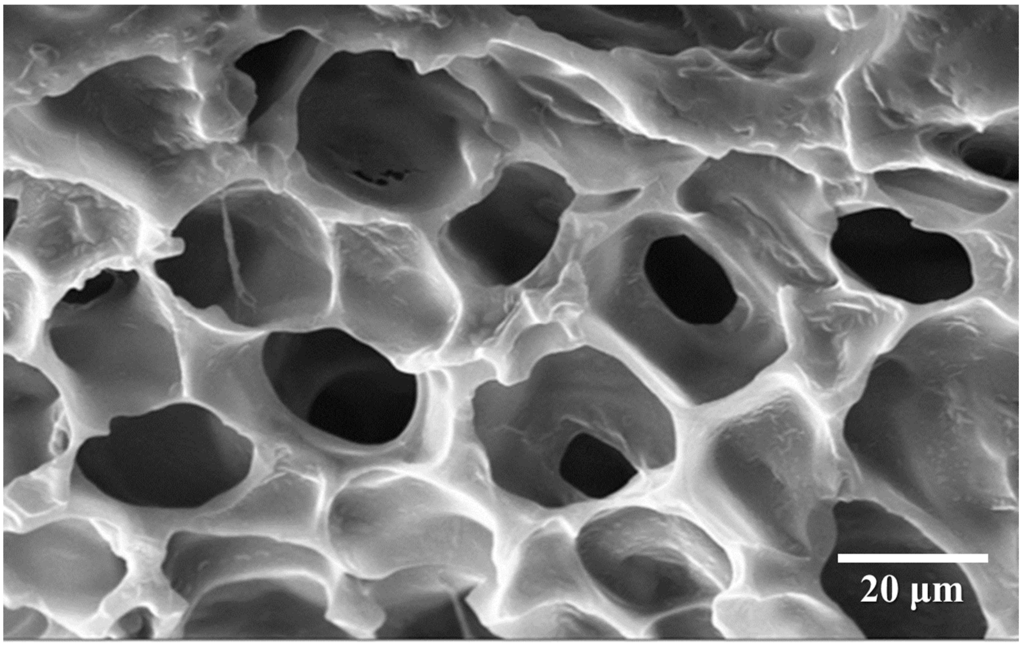

Supplementary Figure S1. SEM imageof PVA-ATMP hydrogels.

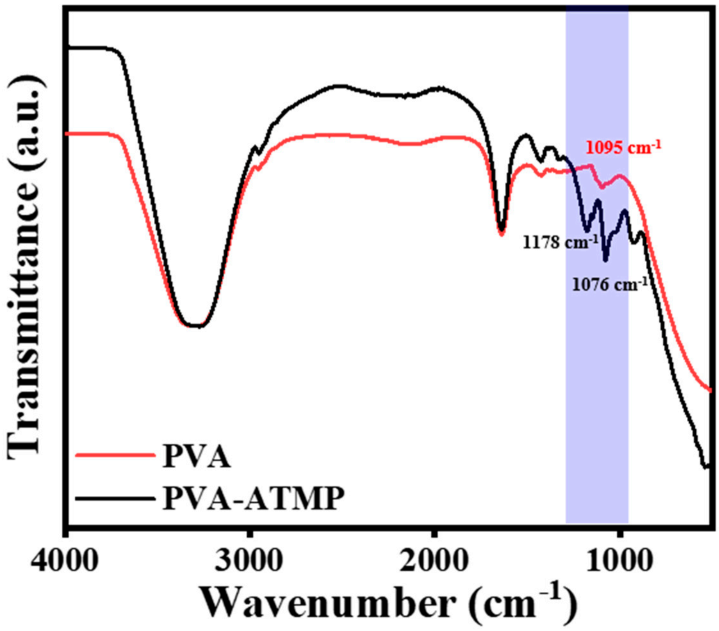

Supplementary Figure S2. Normalized comparative FTIR spectra of pure PVA gel and PVA-ATMP gel.

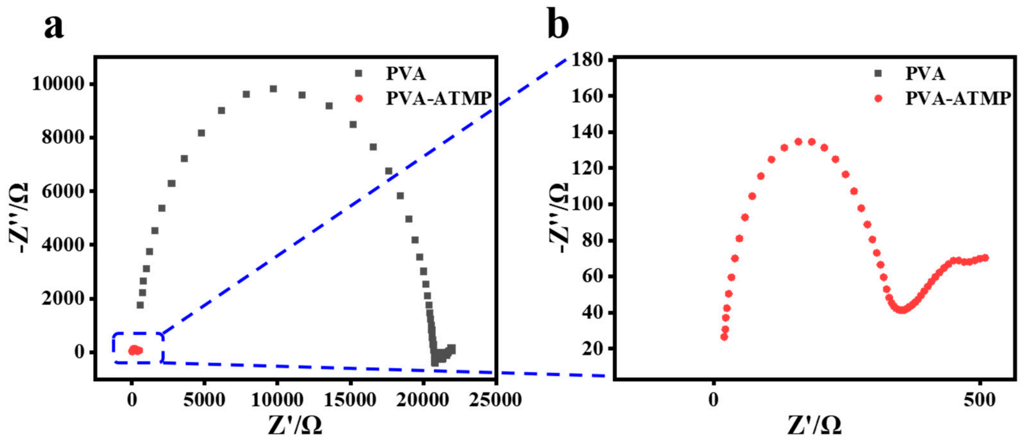

Supplementary Figure S3. EIS Spectra of PVA and PVA-ATMP Hydrogels.

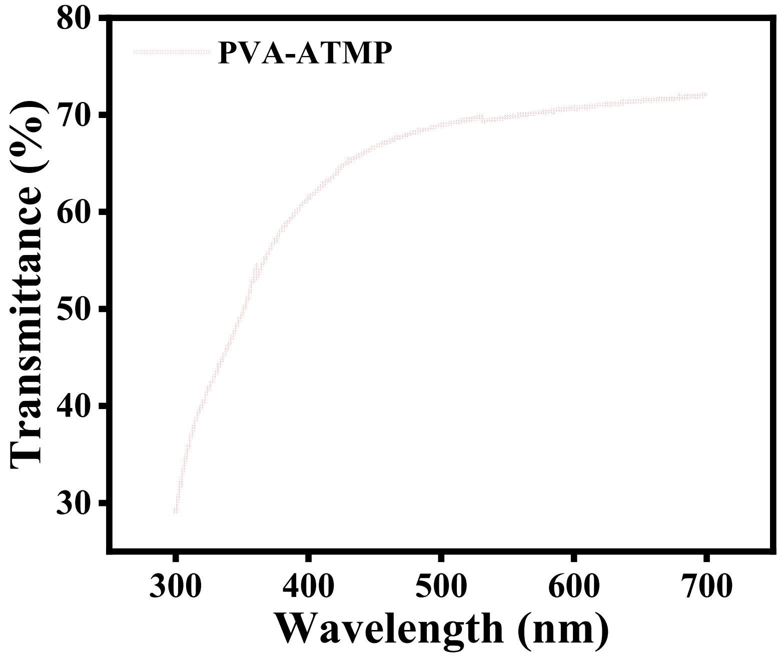

Supplementary Figure S4. The UV–Visible Transmittance Spectrum of PVA-ATMP Hydrogel.

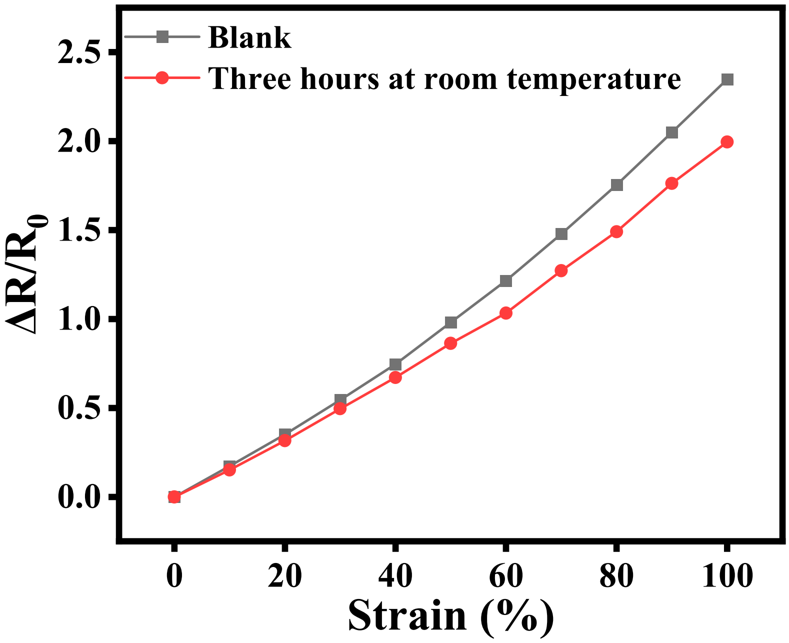

Supplementary Figure S5. Short-Term Dehydration Resistance of PVA-ATMP Hydrogel.

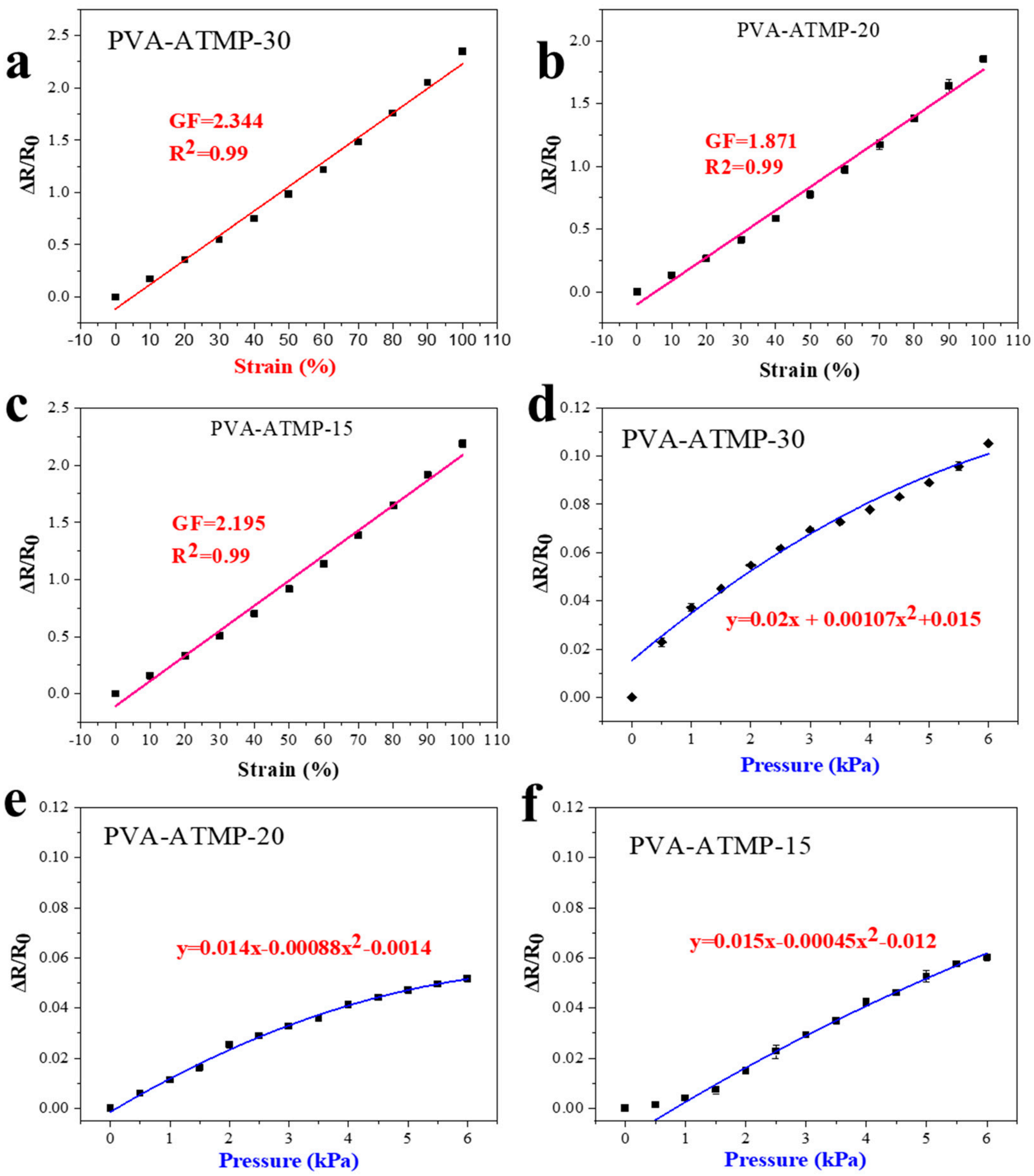

Supplementary Figure S6. (a-c) Tensile resistance-strain curves of PVA-ATMP hydrogels with different ratios; (d-f) Corresponding compression resistance - strain curves.

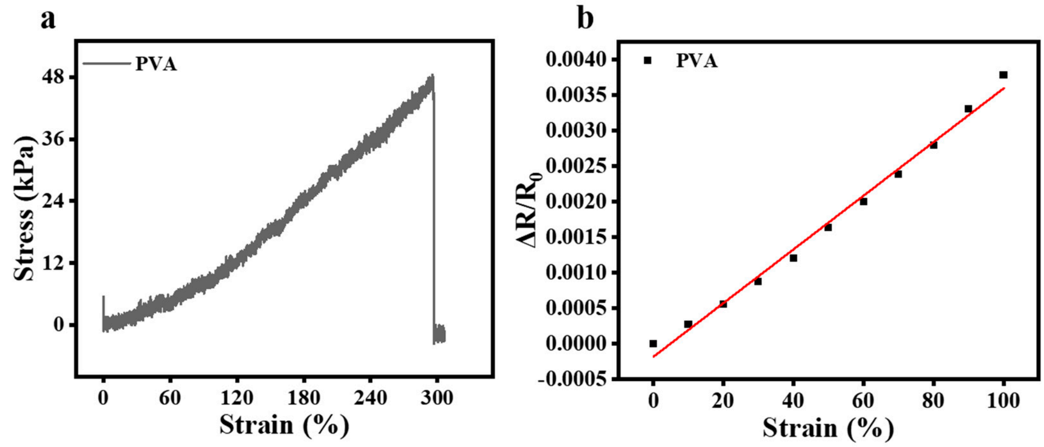

Supplementary Figure S7. Performance Characteristics of Pure PVA Hydrogel.

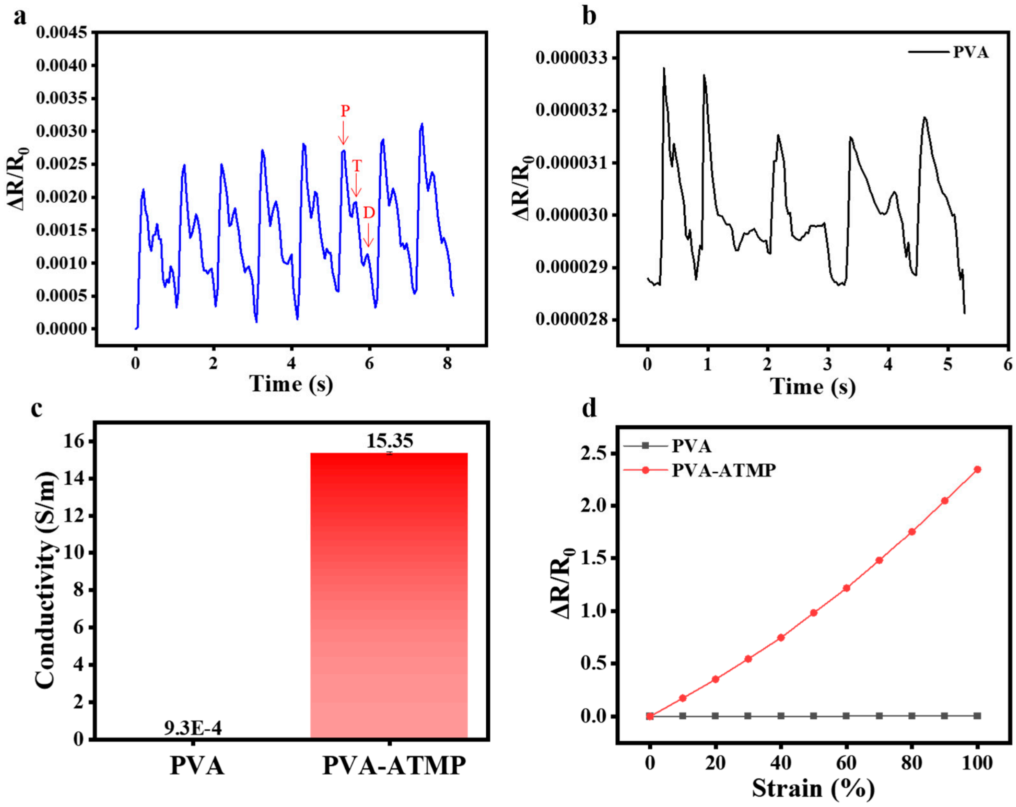

Supplementary Figure S8. Performance Comparison Between PVA and PVA-ATMP Hydrogels.

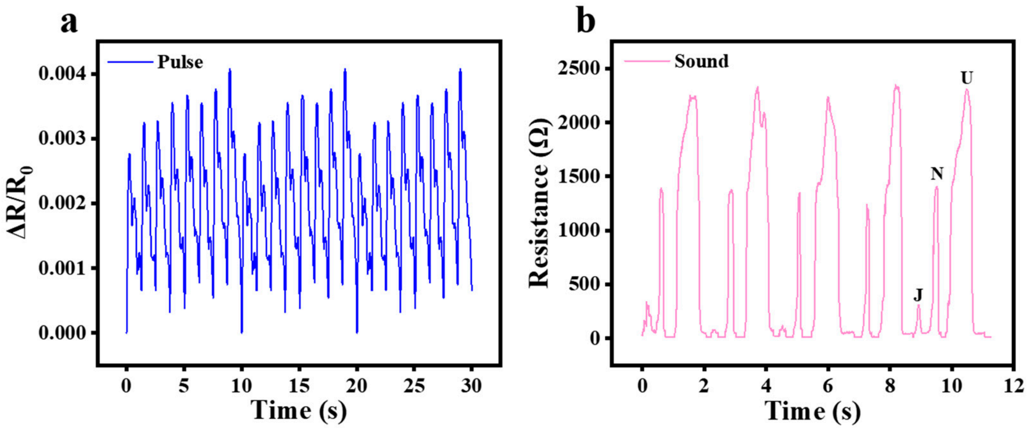

Supplementary Figure S9. Real-time repeatability of diverse human physiological signals.

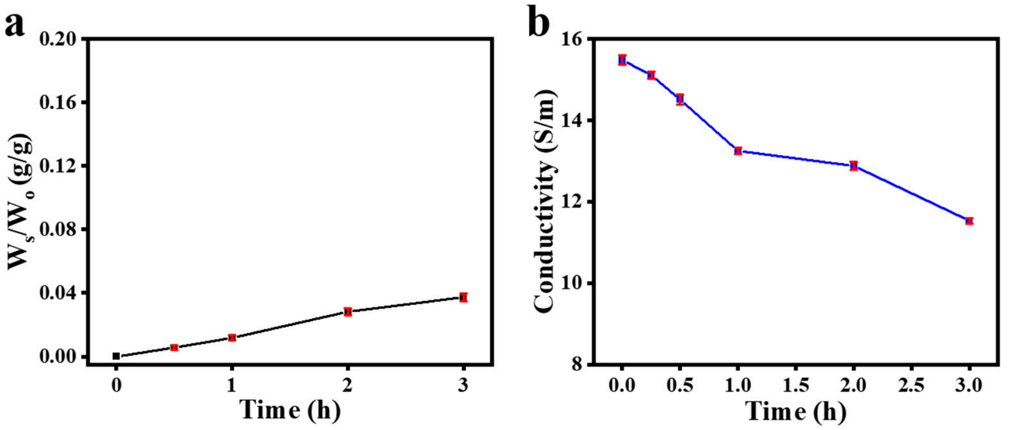

Supplementary Figure S10. Environmental Stability of PVA-ATMP Hydrogel.

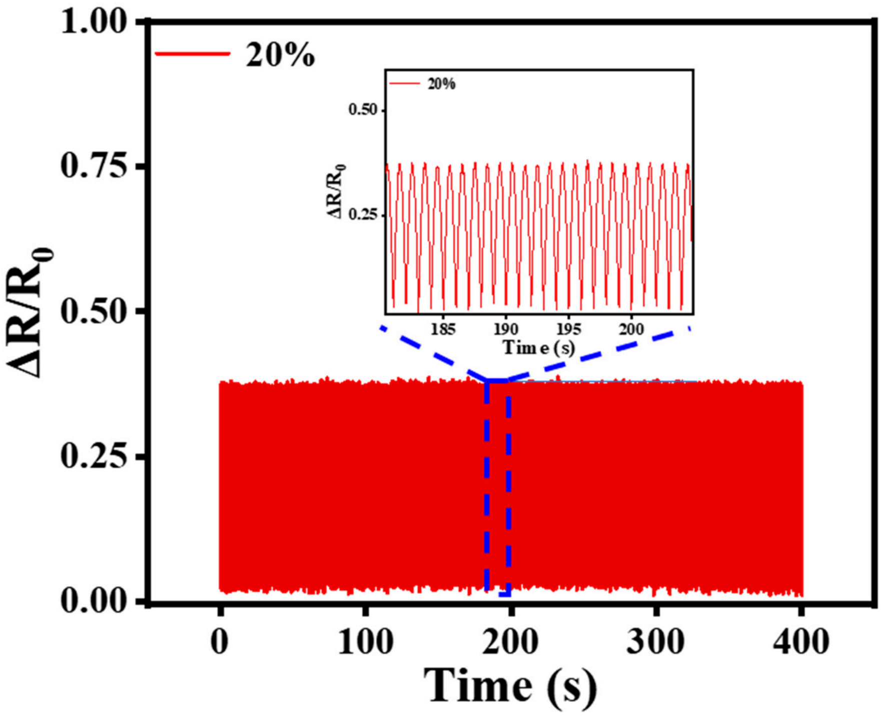

Supplementary Figure S11. Cyclic Response of PVA-ATMP Hydrogel under 20% Strain for 200 Stretch–Release Cycles.

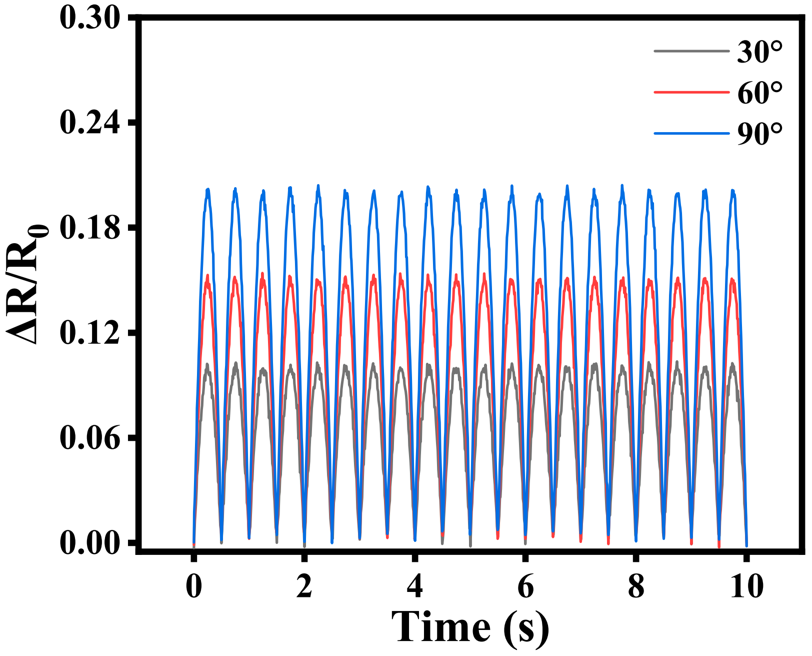

Supplementary Figure S12. Dynamic electrical response of PVA-ATMP hydrogel under different bending angles (30°, 60°, 90°).

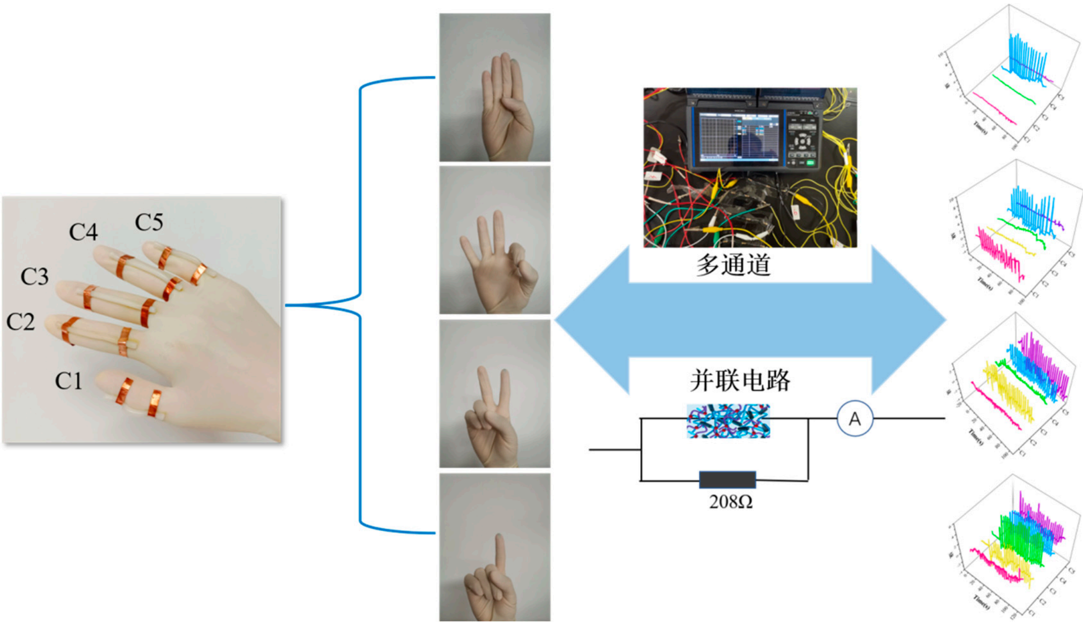

Supplementary Figure S13. System architecture for sensor-based gesture recognition and analysis.

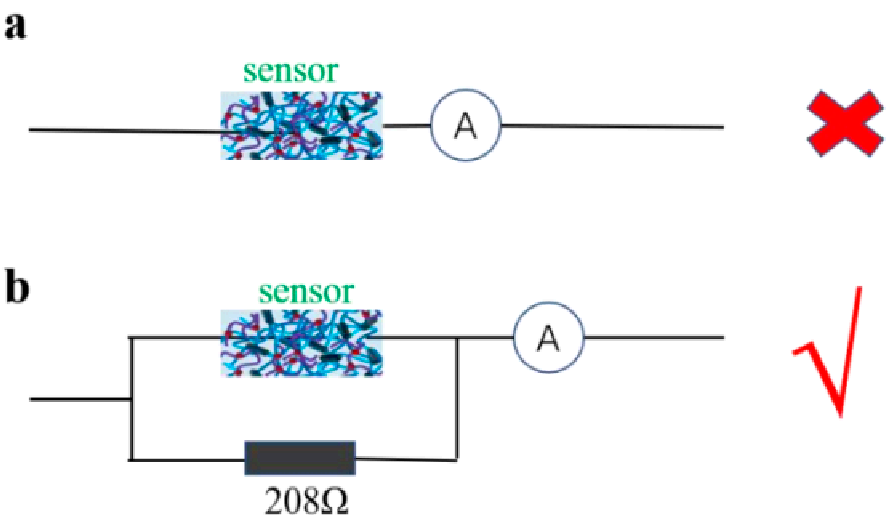

Supplementary Figure S14. Schematic diagram of circuit design of PVA-ATMP hydrogel sensor for complex action analysis. (a)Series circuit; (b) Parallel circuit.

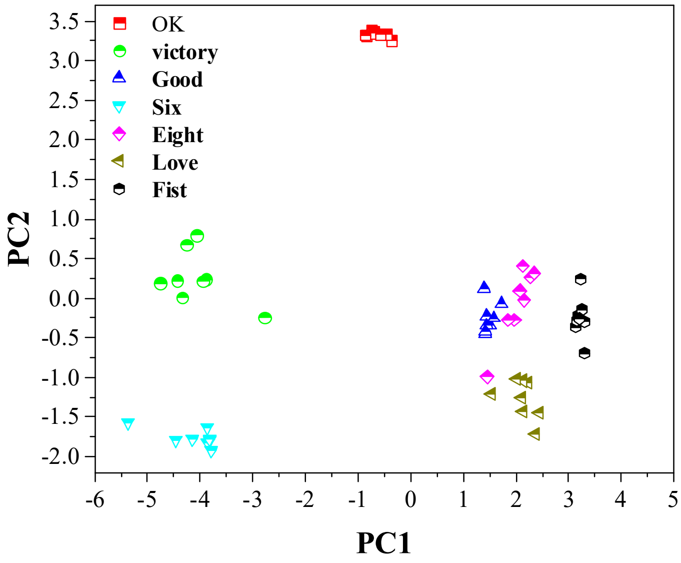

Supplementary Figure S15. The discriminated analysis of seven hand gestures.

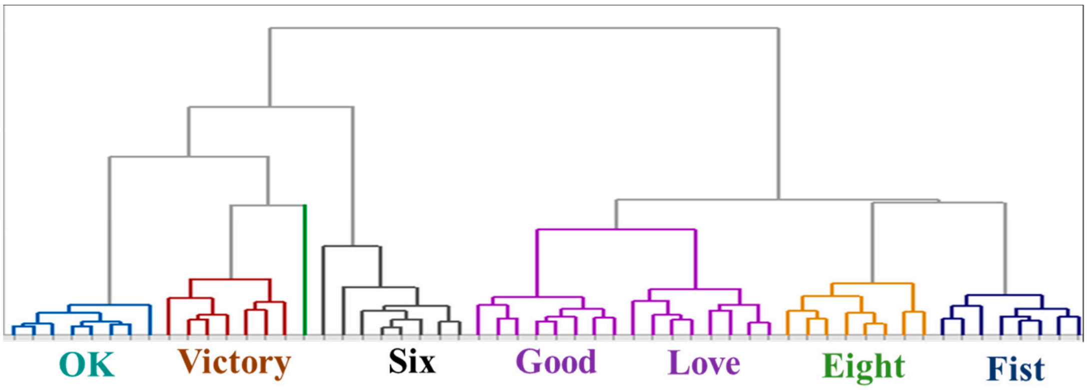

Supplementary Figure S16. HCA score plots for the discrimination of seven hand gestures.

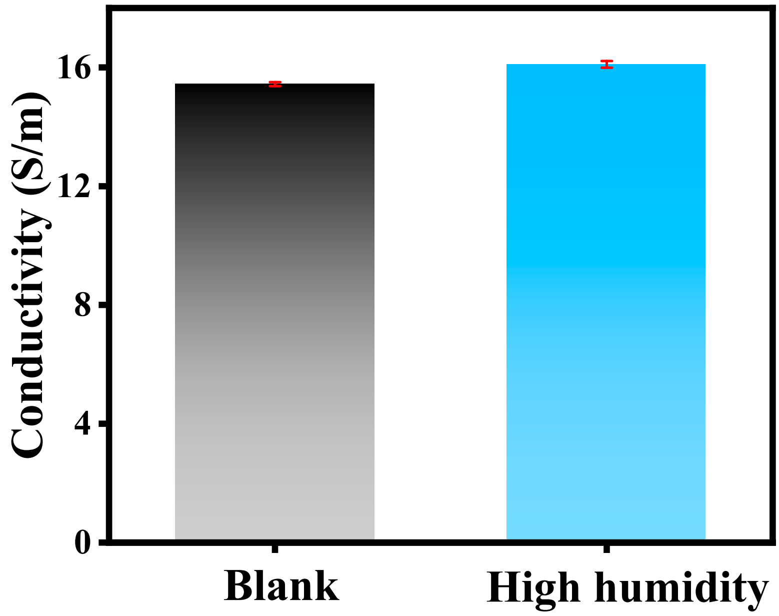

Supplementary Figure S17. Conductivity Variation of PVA-ATMP Hydrogel After 2-Hour Exposure to 90% Relative Humidity.

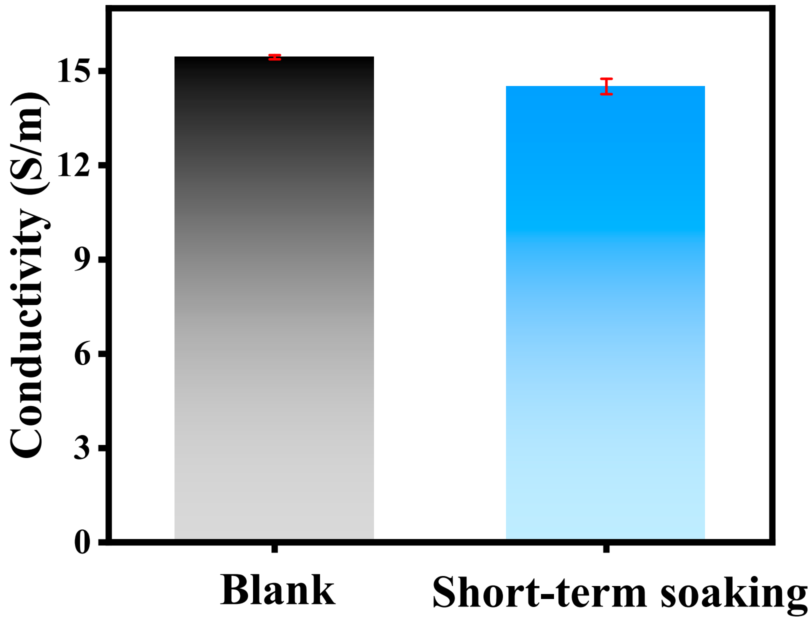

Supplementary Figure S18. Conductivity Variation of PVA-ATMP Hydrogel After Short-Term Immersion in Water.

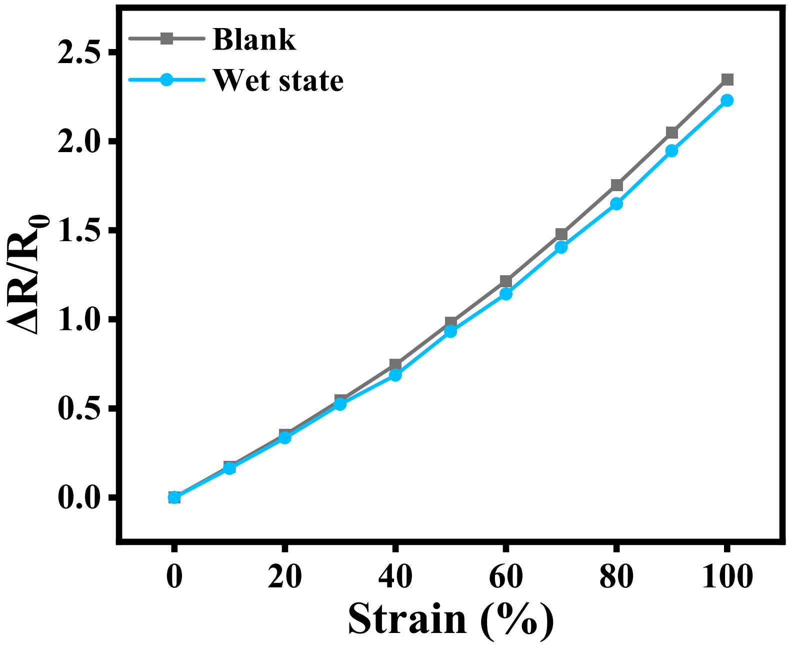

Supplementary Figure S19. Strain Response Variation of PVA-ATMP Hydrogel Under Humid Conditions.

Tables

Supplementary Table S1. Performance comparison between this work and representative hydrogels.

Hydrogel system	Modulus (kPa)	Conductivity (S/m)	GF	Ref.
PVA-Ferritin	112.2	0.15	1.7	[3]
PVA-PEDOT-PSS	5.3	0.5	1.5	[4]
PVA-CNF-ZnSO4	4	0.32	1.7	[5]
PVA-NaCl	1000	7.14	0.989	[6]
PVA-HPC-NaCl	590	2.75	1.31	[7]
PVA/FeCl3/CNF/CNT	119	0.57	1.39	[8]
PVA/TA/CaCl2	430	1.3	2.96	[9]
PVA/Go/NaCl	0.2	3.38	2.05	[10]
P(ATAC–HEMA)	73.29	0.00421	0.205	[11]
**This Work**	**730**	**15.43**	**2.34**	

Supplementary Table S2. Comparison of High-Stretchability DN Hydrogels vs. PVA-ATMP.

Hydrogel System	Strain at Break (%)	Tensile Modulus (kPa)	Crosslinking Mechanism	Self-Recovery/Repeatability	Synthesis Complexity	Ref.
PAMPS/PAAm	800-1200	800-1500	Covalent (sacrificial) + physical	Limited	High	[12]
PAAm-PEGDA DN Hydrogel	400-1500	500-1200	Covalent (PEGDA) + physical	Poor	High	[13]
PAM/PAA DN Hydrogel	600-1000	600-1300	Covalent + ionic	Moderate	Moderate	[14]
Alginate/PAAm DN Hydrogel	700-1100	400-900	Ionic (alginate) + covalent	Moderate	Moderate	[15]
**PVA-ATMP (This Work)**	**1900**	**12-730**	**Physical (dynamic hydrogen bonds)**	**Excellent**	**Low**	

Supplementary Table S3. Performance Comparison Between Pure PVA and PVA-ATMP Hydrogels.

	Modulus (kPa)	Conductivity (S/m)	GF
PVA	12.25	0.000934	$3.78*10^{-5}$
PVA-ATMP	730	15.43	2.34

Supplementary Table S4. Comparison of High-GF Hydrogel Strain Sensors.

Hydrogel System	GF	Strain Range (%)	Response Linearity	Structural Mechanism	Reversibility	Ref.
MXene-PAM Hydrogel	290.96	0-100	Low	Nanofiller-enhanced; MXene layers	Low	[16]
Graphene Hydrogel	20-40	0-20	Low	Crack expansion & graphene dispersion	Low	[17]
Crack-Engineered Hydrogel	59.7	0-100	Low	Microcrack propagation	Low	[18]
PEDOT:PSS-PVA	3.18	0-200	Moderate	Nanofiller & hydrogen bonding	Moderate	[4]
**PVA-ATMP (This Work)**	**2.34**	**0-110**	**High**	**Dynamic hydrogen bonding only**	**Excellent**	

Supplementary Table S5. Results of the LDA of seven hand gestures.

Jackknifed Classification Matrix

	A1	A2	A3	A4	A5	A6	A7	%correct
A1	8	0	0	0	0	0	0	100
A2	0	8	0	0	0	0	0	100
A3	0	0	8	0	0	0	0	100
A4	0	0	0	8	0	0	0	100
A5	0	0	0	0	8	0	0	100
A6	0	0	0	0	0	8	0	100
A7	0	0	0	0	0	0	8	100
Total	8	8	8	8	8	8	8	100

Supplementary Table S6. Comparison of the conductivity and Maximum tensile strength of our Multi-Functional Sensors with Existing ion gel sensors.

**Type of device**	**conductivity** (S/m)	**Maximum tensile strength**(MPa)	**Ref.**
resistance	12.50	0.16	[19]
resistance	6.67	0.025	[20]
resistance	1.50	0.70	[21]
resistance	4.85	0.080	[22]
resistance	4.96	0.49	[23]
resistance	0.43	0.29	[24]
resistance	1.47	0.07	[25]
resistance	0.86	0.56	[26]
resistance	6.25	0.19	[27]
resistance	7.85	0.14	[28]
resistance	2.88	0.80	[29]
resistance	0.34	0.087	[30]
resistance	15.43	0.70	**This work**

References

1. Zhao, S.; Zhang, B.; Yang, J.; Zhou, J.; Xu, Y. Linear discriminant analysis. *Nat. Rev. Methods Primers* **2024**, *4*, 70.
2. Huang, Y.; Li, F.Y.; Qin, M.; Jiang, L.; Song, Y.L. A Multi-stopband Photonic-Crystal Microchip for High-Performance Metal-Ion Recognition Based on Fluorescent Detection. *Angew. Chem. In. Ed.* **2013**, *52*, 7296-7299.
3. Fu, Q.; Tang, J.; Wang, W.; Wang, R. Biocomposite Polyvinyl Alcohol/Ferritin Hydrogels with Enhanced Stretchability and Conductivity for Flexible Strain Sensors. *Gels* **2025**, 11, 59.
4. Cao, J.; Zhang, Z.; Li, K.; Ma, C.; Zhou, W.; Lin, T.; Xu, J.; Liu, X. Self-Healable PEDOT:PSS-PVA Nanocomposite Hydrogel Strain Sensor for Human Motion Monitoring. *Nanomaterials* **2023**, 13, 2465.
5. Hu, J.; Wu, Y.; Yang, Q.; Zhou, Q.; Hui, L.; Liu, Z.; Xu, F.; Ding, D. One-pot Freezing-thawing Preparation of Cellulose Nanofibrils Reinforced Polyvinyl Alcohol Based Ionic Hydrogel Strain Sensor for Human Motion Monitoring. *Carbohyd. Polym.* **2021**, 275, 118697.
6. Di, X.; Ma, Q.; Xu, Y.; Yang, M.; Wu, G.; Sun, P. High-performance ionic conductive poly(vinyl alcohol) hydrogels for flexible strain sensors based on a universal soaking strategy. *Mater. Chem. Front.* **2021**, 5, 315-323.
7. Zhou, Y.; Wan, C.; Yang, Y.; Yang, H.; Wang, S.; Dai, Z.; Ji, K.; Jiang, H.; Chen, X. Highly Stretchable, Elastic, and Ionic Conductive Hydrogel for Artificial Soft Electronics. *Adv. Funct. Mater.* **2018**, 29, 1806220.
8. Li, Y.; Ren, P.; Sun, Z.; Xue, R.; Ding, D.; Tian, W.; Ren, F.; Jin, Y.; Chen, Z.; Zhu, G. High-strength, anti-fatigue, cellulose nanofiber reinforced polyvinyl alcohol based ionic conductive hydrogels for flexible strain/pressure sensors and triboelectric nanogenerators. *J. Colloid. Interf. Sci.* **2024**, 669, 248-257.
9. Liu, Y.; Liu, R.; Liu, H.; Li, D.; Fu, S.; Jin, K.; Cheng, Y.; Fu, Z.; Xing, F.; Tian, Y. Tough, high conductivity pectin polysaccharide-based hydrogel for strain sensing and real-time information transmission. *Int. J. Biol. Macromol.* **2024**, 257, 128575.
10. Wei, J.; Wang, R.; Pan, F.; Fu, Z. Polyvinyl Alcohol/Graphene Oxide Conductive Hydrogels via the Synergy of Freezing and Salting Out for Strain Sensors. *Sensors* **2022**, 22, 3015.
11. Filipecka-Szymczyk, K.; Makowska-Janusik, M.; Marczak, W. Molecular Dynamics Simulations of HEMA-Based Hydrogels for Ophthalmological Applications. *Molecules*. **2024**, 29, 5784.
12. Gong, J.; Katsuyama, Y.; Kurokawa, T.; Osada, Y. Double-Network Hydrogels with Extremely High Mechanical Strength. *Adv. Mater.* **2003**, 15, 1155-1158.
13. Zhu, R.; Zhu, D.; Zheng, Z.; Wang, X. Tough double network hydrogels with rapid self-reinforcement and low hysteresis based on highly entangled networks. *Nat. Commun.* **2024**, 15, 1344.
14. Zhu, L.; Qiu, J.; Sakai, E. A high modulus hydrogel obtained from hydrogen bond reconstruction and its application in vibration damper. *RSC Adv.* **2017**, 7, 43755-43763.
15. Sun, J.; Zhao, X.; Illeperuma, W.; Chauduri, O.; Oh, K.; Mooney, D.; Vlassak, J.; Suo, Z. Highly stretchable and tough hydrogels. *Nature* **2012**, 489, 133–136.
16. She, W.; Shen, C.; Xue, Z.; Zhang, B.; Zhang, G.; Meng, Q. Hydrogel Strain Sensors for Integrating Into Dynamic Organ-on-a-Chip. *Small* **2025**, 21, e2407704.
17. Lv, J.; Kong, C.; Yang, C.; Yin, L.; Jeerapan, I.; Pu, F.; Zhang, X.; Yang, S.; Yang, Z. Wearable, stable, highly sensitive hydrogel–graphene strain sensors. *Beilstein J. Nanotechnol.* **2019**, 10, 475–480.
18. Zhou, Y.; Lian, H.; Li, Z.; Yin, L.; Ji, Q.; Li, K.; Qi, F.; Huang, Y. Crack engineering boosts the performance of flexible sensors. *VIEW*. **2022**, 3, 20220025.
19. Zhou, Y.H.; Fei, X.; Tian, J.; Xu, L.Q.; Li, Y. A ionic liquid enhanced conductive hydrogel for strain sensing applications. *J. Colloid Interface Sci.* **2022**, *606*, 192-203.

20. Zheng, Z.; Xu, W.; Wang, Y.; Xiong, W.; Xiong, C.; You, L.; Wang, S. High-conductivity and long-term stability strain sensor based on silk fibroin and polyvinyl alcohol hydrogels. *Mater. Today Commun*. **2024**, *38*, 108465.
21. Wang, H.; Lin, H.; Hu, X.; Zhou, Z.; Chen, Q.; Hong, M.; Fu, H. Highly Flexible, Freezing-Resistant, Anisotropically Conductive Sandwich-Shaped Composite Hydrogels for Strain Sensors. *Ind. Eng. Chem. Res*. **2023**, *62*, 5563–5573.
22. Zhao, K.; Zhao, Y.; Xu, J.; Qian, R.; Yu, Z.; Ye, C. Stretchable, adhesive and self-healing conductive hydrogels based on PEDOT:PSS-stabilized liquid metals for human motion detection. *Chem. Eng. J*. **2024**, *494*, 152971.
23. Yu, Y.; Zhou, Z.; Ruan, H.; Li, Y. High conductivity, low-hysteresis, flexible PVA hydrogel multi-functional sensors: Wireless wearable sensor for health monitoring. *Chem. Eng. J*. **2025**, *505*, 158877.
24. Jiang, Z.Q.; Shi, X.Y.; Qiao, F.H.; Sun, J.Z.; Hu, Q.L. Multistimuli-Responsive PNIPAM-Based Double Cross-Linked Conductive Hydrogel with Self-Recovery Ability for Ionic Skin and Smart Sensor. *Biomacromolecules* **2022**, *23*, 5239-5252.
25. Zhang, X.; Zhang, J.; Liao, W.; Zhang, D.; Dai, Y.; Wu, C.; Wen, J.; Zeng, W. Stretchable conductive hydrogels integrated with microelectronic devices for strain sensing. *J. Mater. Chem. C* **2023**, *11*, 15873.
26. Li, M.; Yang, Y.; Yue, C.; Song, Y.; Manzo, M.; Huang, Z.; Cai, L. Stretchable, sensitive, and environment-tolerant ionic conductive organohydrogel reinforced with cellulose nanofibers for human motion monitoring. *Cellulose* **2022**, *29*, 1897–1909.
27. Tian, X.; Niu, B.; Hua, T.; Yang, M.Y.; Yang, Y.Y.; Dong, S.S. ontinuous Fabrication of a Highly Integrated, User-Friendly, and Low-Cost Triboelectric Yarn/Fabric for Diverse Sensing Applications. *ACS Sustainable Chem. Eng*. **2023**, *11*, 16087-16097.
28. Qin, Z.H.; Liu, S.D.; Bai, J.H.; Yin, J.J.; Li, N.; Jiao, T.F. Ionic conductive hydroxypropyl methyl cellulose reinforced hydrogels with extreme stretchability, self-adhesion and anti-freezing ability for highly sensitive skin-like sensors. *In. J. of Biol. Macromol*. **2022**, *220*, 90-96.
29. Jin, S.; Jiang, H.; Li, G.; Fu, B.; Bao, X.; Wang, Z.; Hu, Q. Stretchable, conductive PAni-PAAm-GOCS hydrogels with excellent mechanical strength, strain sensitivity and skin affinity. *Chem. Eng. J*. **2020**, *394*, 124901.
30. Cao, Q.; Shu, Z.; Zhang, T.; Ji, W.; Chen, J.; Wei, Y. Magnetic nanocomposite hydrogel with ultra-stretchable as strain sensors for monitoring human motion and the change of magnetic field. *J. Appl. Polym. Sci*. **2024**, *141*, e54934.
